# Recent trends in assistive technology for mobility

**DOI:** 10.1186/1743-0003-9-20

**Published:** 2012-04-20

**Authors:** Rachel E Cowan, Benjamin J Fregly, Michael L Boninger, Leighton Chan, Mary M Rodgers, David J Reinkensmeyer

**Affiliations:** 1 Department of Neurological Surgery, The Miami Project to Cure Paralysis, University of Miami Miller School of Medicine, 1095 NW 14th Terrace, Miami, FL, 33136, USA; 2 Department of Mechanical & Aerospace Engineering, University of Florida, 231 MAE-A Building, P.O. Box 116250, Gainesville, FL, 32611, USA; 3 Department of Physical Medicine & Rehabilitation, University of Pittsburgh School of Medicine, 3471 5th Ave, Suite 201, Pittsburgh, PA, 15260, USA; 4 Rehabilitation Medicine Department, NIH Clinical Center, 10 Center Drive, Bethesda, MD, 20892, USA; 5 Department of Physical Therapy and Rehabilitation Science, University of Maryland, 100 Penn Street, Baltimore, MD, 21201, USA; 6 National Institute of Biomedical Imaging and Bioengineering/NIH, 6707 Democracy Blvd, Bethesda, MD, 20892-5477, USA; 7 Department of Mechanical & Aerospace Engineering, Department of Anatomy and Neurobiology, Department of Biomedical Engineering, University of California, 4200 Engineering Gateway, Irvine, CA, 92697-3875, USA; 8 Human Engineering Research Laboratories, VA Pittsburgh Healthcare System, 6425 Penn Avenue, Suite 400, Pittsburgh, PA, 15206, USA

**Keywords:** Disability, Assistive technology, Robotics

## Abstract

Loss of physical mobility makes maximal participation in desired activities more difficult and in the worst case fully prevents participation. This paper surveys recent work in assistive technology to improve mobility for persons with a disability, drawing on examples observed during a tour of academic and industrial research sites in Europe. The underlying theme of this recent work is a more seamless integration of the capabilities of the user and the assistive technology. This improved integration spans diverse technologies, including powered wheelchairs, prosthetic limbs, functional electrical stimulation, and wearable exoskeletons. Improved integration is being accomplished in three ways: 1) improving the assistive technology mechanics; 2) improving the user-technology physical interface; and 3) sharing of control between the user and the technology. We provide an overview of these improvements in user-technology integration and discuss whether such improvements have the potential to be transformative for people with mobility impairments.

## Introduction

Mobility encompasses an individual’s ability to move his or her body within an environment or between environments and the ability to manipulate objects. Collectively, these activities enable the individual to pursue life activities of their choosing. An individual’s ability to perform any mobility task can be compromised by impaired body functions or structures. Impairments can onset gradually, as occurs with multiple sclerosis, or they can begin instantly, as occurs with traumatic spinal cord injury, cerebral vascular accidents, and limb amputations. The link between impairment and restricted mobility is evident for amputations and spinal cord injury. However, mobility is also affected by less obvious impairments. For example, the pain associated with knee osteoarthritis can significantly affect walking ability. Persons with reduced heat tolerance, such as those with multiple sclerosis, experience decreased endurance and increased fatigue as ambient temperature increases [1]. Regardless of which body structure or function is impaired, technology can improve mobility. Wheelchairs, walking aids, and prosthetic limbs are examples of technologies that have provided widespread benefit.

To identify new opportunities for improving assistive technologies for persons with a mobility impairment, the National Science Foundation initiated a study using the World Technology Evaluation Center. A scientific panel of national experts was formed and charged with gathering information about research trends in technology that could transform mobility for people with mobility disabilities. Information gathering involved a 5-day visit by two teams to several of the leading European laboratories working in this area. Given the many pathways by which disability can impact mobility, and given the large number of possible technological solutions, the panel focused on seven mobility-based tasks: posture, balance and transfers, manipulation, walking, stair climbing, other locomotion tasks, and using transportation. Even within this limited scope, the technologies reviewed were not exhaustive, but they did provide insight into some important themes in assistive technology research.

**Figure 1:**
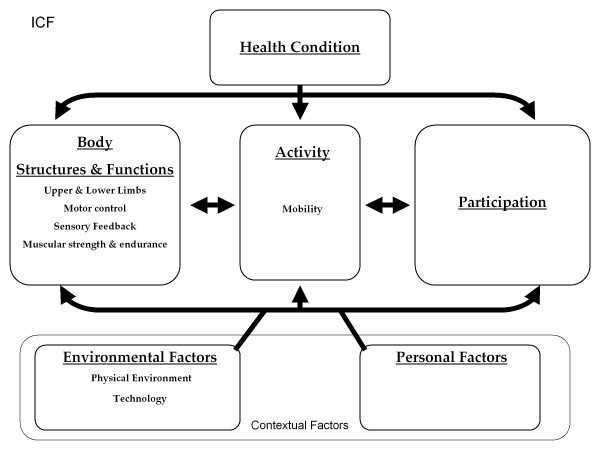
**ICF Framework: Body functions are physiological functions of body systems (including psychological functions).** Body structures are anatomical parts of the body such as organs, limbs, and their components. Impairments are significant deviations from normal or loss of body function or structures. An activity is the execution of a task or action by an individual. Participation is involvement in a life situation. Activity limitations are difficulties an individual has in executing activities. Participation restrictions are problems an individual experiences in involvement in life situations. Environmental factors make up the physical, social, and attitudinal environment in which people live and conduct their lives.

**Figure 2:**
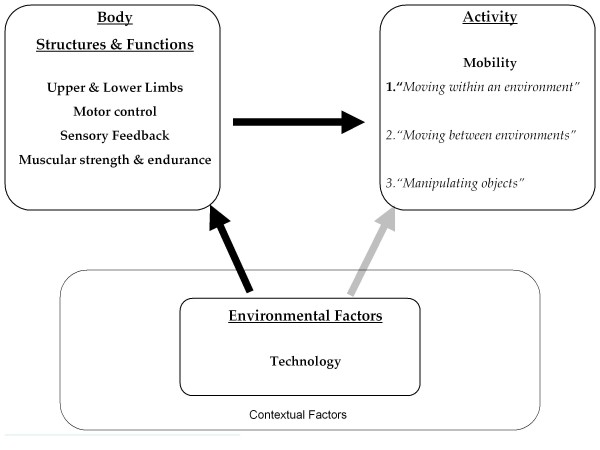
**Simplified ICF framework demonstrating indirect (therapeutic) pathways (black arrows) and direct (assistive) pathways (light arrow) by which technology can improve mobility.** (Modified from World Health Organization model [3]).

Before describing these trends, we briefly discuss a framework for understanding different types of assistive technology. Although there are several frameworks that conceptualize disability [2], the international standard is the World Health Organization’s International Classification of Functioning, Disability and Health (ICF) framework (Figure 1). Like other frameworks, the ICF framework acknowledges that ’disability’ results from the dynamic interaction of the user, technology, and the environment. When environmental demands exceed an individual’s mobility resources, participation may be restricted. Technology can facilitate participation by indirectly (via treatment or therapy) or directly (via physical assistance) enhancing and individual’s mobility such that their mobility capacity meets or exceeds the demand of the environment (Figure 2).

Indirect, or therapeutic, technologies enhance mobility by reducing impairments at the body structure/function level by helping the body in repairing or redressing the body structure impairment, or by supporting rehabilitation of the impaired body function (Figure 2, black arrows). Baclofen pumps are an example of an indirect approach because they facilitate mobility by allowing a person to control his or her spasticity. Robotic therapy devices are another example of an indirect approach because they allow people to reduce impairment through repetitive movement training. Therapeutic technologies typically require clinical oversight to be set-up and operated, are one modality in an overall rehabilitation plan, and are typically not designed to be used to execute daily activities outside the clinic. A companion article reviews recent advances in therapeutic technologies [4].

On the other hand, direct, or assistive, technologies (AT) enhance mobility without altering the impaired body structure/function (Figure 2, grey arrow). Wheelchairs and walkers are prime examples; they enhance mobility, but they do not alter the impairment underlying the mobility loss. Direct technological approaches can augment or support impaired body structure or function, as in the case of a cane or walker, or they can replace the missing or impaired body structure or function, as in the case of a prosthetic limb. In contrast to therapeutic technologies, assistive technologies are operated by the user rather than a clinician and they are designed to be used to execute functional activities in the home and community. The focus of this article is on recent trends in direct technology or AT approaches to enhancing mobility.

### Recent trends in assistive technology for mobility: improved user-technology integration

As stated in the abstract, the unifying theme or trend of the research we observed is a more seamless integration of the capabilities of the user and the assistive technologies. The observed approaches to enhance integration can be broadly classed into three non-mutually exclusive areas; 1) improvements to the assistive technology mechanics; 2) improvements to the user-technology physical interface; and 3) improved shared control between the user and the technology. Improvements in the technology mechanics include hardware and software advances. Improvements to the physical interface typically focused on better leveraging the capabilities of user to operate the technology and providing more intuitive device control.

The panel observed a trend toward better user-technology integration in four key technologies: powered wheelchairs, prosthetics, functional electrical stimulation, and robotic exoskeletons.

### Power wheelchair based mobility

Power wheelchairs are traditionally operated by a joystick and one or more switches which change the function that is being controlled by the joystick. These functions include wheelchair movement, seat tilt, backrest recline, footrest elevation, and seat elevation. Not all persons who could experience increased mobility by using a powered wheelchair possess the necessary cognitive and neuromuscular capacity needed to navigate a dynamic environment with a joystick. For these users, a “shared” control approach coupled with an alternative interface is indicated.

Shared control has been considered before for powered wheelchair mobility [5]. In a traditional shared control system, the assistive technology ‘assists’ the user in path navigation. Shared control systems typically have several modes that vary the assistance provided (i.e., user autonomy) and movement algorithms. Millan et al. suggest shared control approaches can be classified in two ways: 1) mode changes triggered by the user via a button or trigger or 2) mode changes hard-coded to occur when specific conditions are detected [5]. Both approaches have potential problems. Requiring the user to trigger mode changes imparts a substantial mental load, can be tiring, increases complexity, and decreases user-friendliness. Hard coding mode changes may not allow customization to the individual and their specific abilities.

Dr. Etienne Burdet’s Human Robotics research group at Imperial College, in collaboration with the National University of Singapore, has developed a low cost power wheelchair shared control system based on path guidance that provides a third way to address shared control. The target population for the collaborative wheelchair assistant (CWA) is “people who find it difficult or impossible to use a standard power wheelchair but have sufficient sensory abilities to detect when stopping is necessary,” such as persons with cerebral palsy, traumatic brain injury, or locked-in individuals [6]. The CWA guides the user along previously programmed paths between specific destinations. Paths are programmed by a “helper” who walks the chair through the desired pathway while the chair records the path. The user controls speed, starts, and stops, as well as any deviations required to avoid obstacles that have entered the pre-programmed path. However, the burden of navigation falls on the wheelchair, which adheres to the programmed path until the user initiates a deviation. During the deviation, the chair acts like a mass-spring-damper system being pushed away from the pre-programmed path by the user. The wheelchair thus returns to the path once the user relinquishes control. The benefit of this approach is that the cognitive load of navigation and path-planning is not born by the user. The user only needs to focus on obstacle avoidance and speed control. Before users navigate new environments, new paths must be created. It is envisioned that new ‘path libraries’ could be automatically generated from building plans. This shared approach does not fit into the categories defined by Millan et al. [5] as there are no mode changes triggered by the user or via automatic sensing; it is rather an approach that more seamlessly integrates the user and machine.

Other approaches toward improving powered mobility are seeking to exploit better the user’s inherent capabilities for controlling the chair through better input devices. One approach is to design an interface that can be operated by an alternate body part. An example of this approach is the development of tongue based control interfaces, such as that developed by the Sensory Motor Interaction (SMI) center of the department of health science and technology at Aalborg University. It is an inductive system relying on a ferromagnetic tongue piercing and an intraoral device embedded with 18 sensors. Ten sensors are dedicated to a keyboard and eight to joystick control. It has been designed to interface with most power wheelchairs that can be controlled by traditional joysticks [7]. In a related approach, a group at Georgia Institute of Technology (USA) has developed a tongue interface that is not dependent on a physical interface between the tongue and sensors. Instead, sensors external to the oral cavity wirelessly track tongue position via a tongue-mounted magnetic sensor. This interface has been tested in 13 persons with high cervical spinal cord injuries [8]. Other related work has explored how information from sensors placed anywhere on the body can be automatically mapped to wheelchair control signals, again allowing a person to use the parts of the body that they are capable of moving well to control the wheelchair [9].

Another way to better make use of a user’s inherent capabilities is to use a brain computer interface (BCI) to detect, decode, and communicate intended movements from brain electrical activity. A previous NSF study examined recent progress in BCI technology [10] in detail, so we only summarize a few important points here. Current noninvasive BCI technology is characterized by a low information transfer rate (low bandwidth), which is a challenge for real-time wheelchair navigation. Low bandwidths can result in substantial delays between when a user initiates a maneuver and when the wheelchair responds, introducing a potential safety hazard [11]. In addition, BCI driven wheelchair navigation typically requires extensive training, imposes a substantial cognitive load, and can be very tiring. If BCIs are to mature into a realistic option to control power wheelchairs, these issues must be resolved in a cost-effective manner.

Dr. Burdet’s Imperial College group has developed a possible solution to these challenges using the shared control system described previously. The computer “drives” the chair between destinations using pre-programmed paths while the user monitors the pathways for unexpected obstacles. A slow BCI is used for selecting among the destinations and a “fast” one is used for emergency stopping. This approach removes the cognitive load of navigation, preventing the inevitable fatigue, and does not require extensive training, but it limits use of the system to known environments and programmed destinations [11].

### Prosthetic limb control

Prosthetic development challenges include replacing both the efferent nervous system (i.e., movement) and the afferent nervous system (i.e., sensory feedback). Adequate prosthetic limb control will be achieved when both efferent and afferent systems are adequately replaced. Three novel approaches were observed in Europe for better interfacing the user and their prosthetic: 1) computer-vision enhanced control, which is an example of improving both the device and the shared control system, 2) peripheral nervous system interfaces, an example of improved interfaces, and 3) kinematic/kinetic based control, a strategy which improves the mechanics of the limb through software and provides a better interface. The first two approaches target upper limb prosthetic control and the third targets lower limb.

#### Computer-vision enhanced control

When an individual reaches to grab an object, the hand assumes a given orientation and opens to accommodate the object. Typically, prosthetic hand control has a high mental burden, as the user must plan the grasp and generate step by step commands to position and shape the hand. Although a high degree of control can be achieved by this method, users prefer intuitive controls requiring less conscious involvement. In pursuit of a less demanding control strategy, researchers at the University of Aalborg have developed a camera-based shared control system that uses image recognition to autonomously select the proper hand orientation, grasp shape, and grasp size based on images of the object being manipulated [12]. The user is responsible for aiming, triggering, and orienting the hand, while the camera-based control selects and implements grasp type and size. By increasing the autonomy of the prostheses, user burden is lessened. The system was successfully tested in 13 non-disabled subjects who used it to control an artificial hand [13]. Once refined, this system is targeted for application with the Pisa Hand, a product of the European Union funded Smart Hand project.

#### Peripheral nervous system interface (PNS) control

Upper extremity prosthetic control is challenging due to both the number of possible motions to be controlled and the limited number of sites for traditional control interfaces. An appealing option is controlling a prosthetic arm or hand via the same nerve that once carried afferent and efferent information between the arm and brain [14]. Potentially, this approach would be more intuitive to the user and provide a pathway to deliver sensory feedback.

At Scuola Superiore Sant’Anna in Italy, Dr. Silvestro Micera has explored this option by implanting thin-film longitudinal intrafasicular electrodes in the median and ulnar nerve of a trans-radial amputee. The subject was implanted with a prosthetic hand mounted directly to the distal end of the residual radius. During a four week trial, the subject was trained to imagine three distinct hand/finger movements, with the resultant muscle activity being recorded. In addition, the afferent fibers were stimulated to determine if it was possible to deliver sensory feedback to the brain. Dr. Micera concluded that it was possible control at least three different grip types using the neural signals recorded by the PNS interface. He suggests that more grips should be possible but that additional interface electrodes may be required. Finally, although stimulation of the afferent fibers induced phantom tactile sensation during the first few weeks, the response dissipated by the end of the trial. Dr. Micera suggests refinements to the interfacing electrodes may help solve this problem [15]. Following the four week test period, human subject regulations necessitated removal of the prosthetic hand. However, the subject was disappointed that he was not allowed to keep the hand.

A less invasive approach for improving the control of an artificial hand is being pursued by Dr. Peter Veltink, Dr. Hans Rietman, and co-workers at the University of Twente in Enschede, in The Netherlands. Rather than using muscle activity signals from implanted electrodes to control a prosthetic hand, the Myopro Project is pursuing the use of an array of surface electrodes. Traditionally, prostheses controlled by surface electrodes (i.e., myoelectric prostheses) have had limited control ability due to the use of a small number of electrodes. To address this limitation, researchers in Enschede are using a 4 x 10 grid of electrodes distributed across the residual forearm of the amputee, thereby increasing the number of control signals. These control signals are being mapped on a patient-specific basis to 10 hand positions located at the extremes of 5 hand degrees of freedom (e.g., finger flexion-extension, wrist pronation-supination). The mapping is performed based on the same grid of signals collected from the forearms of healthy subjects performing the 10 hand positions. In testing performed thus far, the approach has over 99% accuracy in classifying hand position for healthy subjects and one amputee subject.

#### Kinematic/kinetic control

At ETH Zurich, in the Sensory Motor Systems laboratory, Dr. Heinke Vallery has developed a novel approach to controlling a transfemoral prosthetic leg equipped with a “powered” knee. During walking, joint motions are strongly coupled. Dr. Vallery’s approach, termed complementary limb motion estimation (CLME), exploits the physiological inter-joint couplings of the intact leg to instantaneously determine the motion required of the prosthetic leg. The estimated motion is then used as a reference to drive the motion of the prosthetic limb. An advantage of CLME is that it allows a wide range of movements and the prosthetic limb is intrinsically synchronized with the non-impaired limb. It has been tested on an amputee during treadmill walking and stair ascent/descent [16].

At the University of Twente in Enschede, Dr. Hans Rietman and colleagues are evaluating a microprocessor-controlled prosthetic knee (Rheo knee Ossur) for above-knee amputees [study is ongoing]. A common problem with passive prosthetic knee designs the lack of automatic adaptation to the characteristics of the patient’s walking pattern [17]. To address this problem, microprocessor-controlled knees are developed that uses real-time position and force measurements. The microprocessor analyzes these measurements and then determines the correct actuation moment depending on the phase, speed, and loading within the gait cycle. With this approach, the need to have a mechanism for locking and unlocking the knee during stance phase is eliminated. However it remains important to know what the real clinical benefits of these developments are for patients with transfemoral amputations. Ongoing research is focused on providing proprioceptive feedback to patients and to enhancing prosthetic control. At the other extreme of complexity is a simple robotic hand [18] developed by researchers at Delft University of Technology in the Netherlands. The underactuated robotic hand possesses three fingers controlled by only one motor. The hand has no sensors, with grasping being achieved using a mechanism that distributes contact forces evenly over the three fingers. The hand is capable of grasping objects of various sizes and shapes both firmly (so that they do not drop) and gently (so that they do not break). The hand was created for industrial applications where repetitive human manipulation is currently required (e.g., packaging of bell peppers). However, it could be used equally well as an assistive device for individuals with limited hand mobility. For example, it could be attached to a wheelchair to provide a versatile option for holding objects of various sizes and shapes, or it could be used to perform a small range of functional tasks such as grasping a door handle to open a door.

### Functional electrical stimulation

Functional electrical stimulation (FES) remains a technology with great potential for restoring movement. Its use is limited in part by the time and effort required to don the systems. A possible solution is to implant parts of the system. Neurodan, building on cuff electrode technology developed at the University of Aalborg, has developed one such solution, called Actigait. An example of a fully implantable solution is the Neurostep, which also times muscle stimulation based on sensing limb state from afferent activity in peripheral nerves. Neurostep relies on both an enhanced interface and improved mechanics as specified by the control software.

Another challenge with FES systems is controlling a large number of degrees of freedom in a way that can achieve functional ambulation and functional use of the upper extremity. By focusing on a non-conventional but large population of potential users, The TREMOR European project is attempting to develop a more widely used multi-channel upper extremity FES system. Tremor is the most common movement disorder, becomes more common with advancing age, and typically affects the upper extremity. Upper extremity tremors can make eating, drinking, or other reaching, grasping, and fine motor tasks extremely difficult. Drugs, surgery, and deep brain stimulation are effective treatment for 75% of cases. To provide treatment for the remaining 25%, the European union has funded the TREMOR project to develop a functional electrical stimulation (FES) orthotic [19]. This orthotic will automatically detect and suppress the tremor by canceling it with out-of-phase muscle stimulation or by co-contraction to stabilize the limb. In this scenario, excessive, rhythmic muscle activity (tremor) is the body impairment and reaching/grasping/eating is the limited activity. The innovation of this technology AT is to improve the user’s inherent reaching/grasping/fine motor activity by removing a superimposed impairment.

### Robotic exoskeletons

Robotic exoskeleton research and development was originated by the military in the 1960’s. However, mobility for people with a disability has recently become a focus of robotic exoskeleton research. As reviewed in a companion paper [4], initial work has focused on therapeutic applications of robotic exoskeletons, with a prime example being the Lokomat gait training robot. Attention is now increasing toward assistive technology applications of robotic exoskeletons in which the exoskeleton is designed to promote functional activities in the home and community.

A recent review identified four characteristics which robotic exoskeletons must embody if their maximal rehabilitative and assistive technology potential is to be realized: 1) robust human-robot multimodal cognitive interaction; 2) safe and dependable physical interaction; 3) true wearability and portability; and 4) user-centered aspects such as acceptance and usability [20]. As apparent from the above discussion of other assistive technologies, the last focus is critical to the success of every assistive technology. If a person cannot easily use or does not accept the assistive technology, he or she will abandon it. AT device abandonment is a well documented phenomenon [21-24] and underlies the emerging awareness that end-users should be involved as soon as possible in the development of assistive technology devices.

A European example of robotic exoskeletons is that of Dr. Jose Pons in Madrid. Dr. Pons has developed an innovative knee-ankle-foot orthosis that can assist people with leg weakness in achieving normal joint kinematics during walking [25]. The normal contribution of the joints to each gait cycle phase is approximated using spring-like, force-length curves. Actuators for each joint are constructed of compression and tension springs. The actuators use solenoids, or an ankle-driven Bowden cable, to reproduce the desired spring characteristics during each phase of the gait cycle. The system has been shown to improve the gait pattern of individuals with poliomyelitis [25,26], and is being investigated for commercialization by Össur.

In the future, the distinction between therapeutic and assistive technologies will dissolve. As robotic exoskeletons advance, patients will be able to wear them in the home and community, receiving both activity-based therapeutic interventions and supportive assistance as needed. This combined assistive-therapeutic model for assistive technology has already been demonstrated for foot drop stimulators. Long-term use of a foot drop stimulator improves the ability of a person to walk, even when the stimulation is turned off [27].

This review does not cover orthopedic implant technology such as total joint replacements. Though such technology has traditionally not been considered to be an assistive technology, it satisfies the traditional definition of assistive technology. Furthermore, it has been one of the most transformative assistive technologies in the past century, allowing millions of people to regain lost function and quality of life. As an example of innovative work in this area observed by the panel, at the Rizzoli Institute in Bologna, Italy, Dr. Alberto Leardini and colleagues have developed a novel total ankle replacement design that is being marketed by an orthopedic implant company. The design maintains natural tension in the ankle ligaments, thereby producing more natural motion and loading profiles than previous total ankle designs [28].

## Discussion

### Factors limiting assistive technology advancements

To recap, the unifying theme of the research we observed was a more seamless integration of the capabilities of the user and the assistive technology. All observed advances were built upon existing assistive technology rather than representing completely new, original, out-side the box technologies. This observation reflects the reality that truly revolutionary, game-changing technology appears very rarely. However, as the below discussion of Oscar Pistorius implies, advances to existing technology can achieve transformative changes in mobility.

Our discussions with our European peers suggest several areas limit our advancement rate. First, the components used to 'build' the assistive technology limits us. Any assistive technology is only as durable, light, and small as the available building blocks. For example, if exoskeletons are to become a viable mobility option for the non-ambulatory, they must be extremely lightweight, and therefore all structural components, actuators, and power sources must decrease in weight. A second core limitation is the relative immaturity of our control algorithms. As an example, using an exoskeleton to recreate the body's ability to move smoothly over varied terrain at varied rates requires intimate access to the user's intended movements, along with sophisticated control algorithms to adjust to the complex, unstable, and varying dynamics of the user’s body and the walking environment. Finally, if the developed technologies are to gain user acceptance and widespread adoption, control interfaces must be intuitive, seamless, and non-obtrusive. Component advancements will achieve seamless and non-obtrusive interfaces. Control algorithm advancements will achieve intuitive control. However, only persons with disabilities can provide design specifications for ‘intuitive’, ‘seamless’, and ‘non-obtrusive’. If we do not make consulting persons with disabilities a priority, we will not meet the demands of the end user, history will repeat itself, and the technology will be abandoned.

### Moving towards transformation?

A key goal of the NSF Study was to identify research themes that could “transform mobility for people with a disability.” It makes sense to ask what transformative technologies will look like, and, more specifically, will improvements in user-technology integration be transformative, or are entirely new technologies required?

To illustrate “transformative,” let us define transformative technology as that which elevates mobility performance by people with a disability to that of their non-disabled peers. An excellent example of this elevation is Oscar Pistorius, a South African paralympic bilateral trans-tibial amputee. With his advanced running prosthesis, Mr. Pistorius competes against non-disabled athletes, winning silver in the 2007 South African 400 m non-disabled track championships and narrowly missing qualification for the 2008 Beijing Olympics. Without his prosthesis, he would not be able to walk, much less compete at an elite level. The transformation provided by the prosthesis and his training is so complete, so dramatic, that for a period, Mr. Pistorius was banned from non-disabled competition because it was thought his prosthesis conferred an illegal performance enhancement. While that ruling has been overturned, the scientific debate continues as to if he has an advantage over his non-disabled competitors [29-32]. Regardless, the gap between what Mr. Pistorius can achieve without his prosthesis and what he achieves with his running prosthesis is transformative.

Mr. Pistorius’ transformation was not made possible by a fundamentally new type of assistive technology; rather an existing technology, a below-knee prosthesis, was designed to have mechanical properties that better integrated with his inherent running ability. Thus, while it is unclear whether the improvements in user-technology integration reviewed here will become transformative, the example of Mr. Pistorius illustrates that the enhanced integration approach itself holds tremendous transformative potential.

Ultimately, while both the form and amount of change required to achieve a transformative improvement will vary according to the degree and type of impairment, the ideal path to quantify “transformative” is to ask the person with the disability. Who better to identify the “what” and “how much” of transformative changes? And yet, research groups and funding agencies have struggled to take this approach toward quantifying transformation. If transformative improvements are the end goal, it is important to develop a user-centered quantification system and to employ it throughout the development process. Ideally, individuals with a disability will invent and refine this quantification system, and furthermore, the required assistive technology themselves – who better? At the very least, continuous end-user involvement will help ensure that developed technologies match user needs and wants, as well as capabilities and impairments.

## Conclusions

The panel saw no fundamentally new assistive technologies on its trip; rather the primary theme in assistive technology development observed was refinement of existing assistive technology in clever ways so that its capabilities integrated better with the user’s capabilities. These refinements are being done on an application-by-application basis through development of improved technology mechanics (e.g., knee-ankle-foot orthosis, kinetic control of a prosthetic limb); improved user interfaces (e.g., tongue or whole-body controllers, electrodes implanted in central and peripheral nervous system) and by automating target control functions in a way that blends the machine’s assistance with the natural abilities of the user. Better integrated control systems decrease user burden, enabling more refined control of highly sophisticated prosthetics or enabling persons with the most severe physical disabilities autonomous mobility in power wheelchairs.

## Competing interests

David Reinkensmeyer has a financial interest in Hocoma, A.G., a company that makes robotic therapy devices. The terms of this arrangement have been reviewed and approved by the University of California, Irvine, in accordance with its conflict of interest policies. The remaining authors declare that they have no competing interest.

## Authors’ contributions

RC was the lead manuscript writer and editor. DR, BF, MB, LC, and MR provided edits and revisions. All authors read and approved the final manuscript.
